# Bio-Activity of Natural Polymers from the Genus *Pistacia:* A Validated Model for Their Antimicrobial Action

**DOI:** 10.5539/gjhs.v4n1p149

**Published:** 2012-01-01

**Authors:** Mohammad Sharif Sharifi, Diako Ebrahimi, David Brynn Hibbert, James Hook, Stuart Loyd Hazell

**Affiliations:** Faculty of Medicine, University of New South Wales Sydney Medical School, University of Sydney, NSW 2006 Australia Tel: 61-421287461 E-Mail: m.sharifi@unsw.edu.au; Faculty of Medicine, University of New South Wales Sydney, NSW 2052, Australia; School of Chemistry, University of New South Wales Sydney, NSW 2052, Australia; Faculty of Sciences, University of Southern Queensland, Australia

**Keywords:** *Pistacia Lentiscose*, Atlantica, *Kurdica*, *Mutica*, *Cabolica*, *Helicobacter Pylori*, Anti-Microbial, GEMANOVA

## Abstract

The polymers from mastic gum of *Pistacia lentiscose* and subspecies of *Pistacia atlantica*, (sp. *kurdica, mutica* and *cabolica*) have been isolated and characterised by gel permeation chromatography (GPC) and ^13^C NMR spectroscopy as *cis*-1,4-poly-β-myrcenes. They were screened against *Helicobacter pylori* and other Gram-negative and Gram-positive bacteria to evaluate their antimicrobial action. In order to further test their hypothesised mode of action, two polymer types were synthesized: one from myrcene, and four from polyvinyl alcohols of different molecular weights, derivatised with p-hydroxybenzoate. The anti-microbial activity of these polymers, evaluated through their ‘kill’ kinetics, was found to be related to their functional groups, their molecular weight and their solubility.

## 1. Introduction

The use of natural and synthetic organic polymers has become the mainstay of many familiar products due to their excellent electrical, mechanical, optical and thermal properties. Natural and synthetic polymers dominate important industries such as rubber, plastics, packaging, specialist coatings, adhesives, medical devices and pharmaceuticals. In pharmaceuticals, polymers are of interest as a means of drug delivery or as a drug itself, e.g., antimicrobials (Batz, Ringsdorf, Ritter, 1974).

The emergence of antibiotic resistance, where bacteria exhibit reduced susceptibility to antimicrobials by mechanisms such as altered drug uptake, drug target alternation and/or drug inactivation, has become a major problem, particularly in hospitals (Cloete, 2003 & Smith, 2005). These mechanisms are undoubtedly significant factors to antibiotic failure in clinical medicine.

One particular problem is where medical devices are contaminated with bacteria growing as adherent biofilms, that is, device-related infections associated with artificial joints or venous catheters. Biofilms are also associated with some other chronic infections, such as those occurring in the respiratory tract (Smith, 2005). To overcome these difficulties, the process of biofilm formation has been studied with a view to preventing or treating infections. (Kerr, Smith, Cowling, Hodgkiess, 2001). Biofilms are targets for “anti-fouling” systems and novel drug delivery technologies (Kerr, *et al.*, 2001). These include surface modification of devices to reduce bacterial attachment and incorporation of antimicrobials (anti-microbial co-polymers) to prevent bacterial colonization (Kalyon & Olgun, 2001). Electronic instruments have also been used either to release antimicrobials from device surfaces or to drive antimicrobials through the biofilm (Smith, 2005). Other technologies include delivery of antibiotics as aerosols to the lungs and formulations as liposomes and polymer-based vehicles (Smith, 2005). Many biodegradable polymer-based carrier systems have also been used, such as poly (lactide-co-glycolide) and thermoreversible hydrogels (Smith, 2005).

Mastic gum, a product of the *Pistachia* genus containing an unusually high molecular weight polymer, *cis*-1,4-poly-β-myrcene (Van den Berg, Van der Horst, Boon, Sudmeijer, 1998), Figure 1, has a long-standing reputation as a healing agent that has only recently been systematically examined for its antimicrobial activity. For example, it has been found to exhibit anti-plaque formation in dental studies, as well as to inhibit *Helicobacter pyrlori*, and other antimicrobial activity (Al-Habbal, Al-Habbal, Huwez, 1984; Ali-Shtayeh & Abu Ghdeib, 1999; Al-Said, Ageel, Parmar. Tariq, 1986; Ebrahimi, Sharifi, Hazell, Hibbert, 2008; Huwez & Al-Habbal, 1986, Maron, Bono, Leone, Bona, Carretto, Perversi, 2001; Paraschos, *et al*. 2007; Sakagami *et al.*, 2009; Sharifi & Hazell, 2009, 2011; Sharifi, Vagg, Hazell, 2001; Zhou *et al.*, 2009).

**Figure 1:**
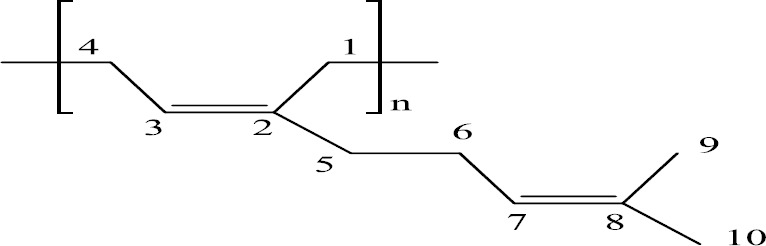
*Cis*-1,4-poly-β-myrcene (polymyrcene)

As a part of our investigation (Ebrahimi, *et al.*, 2008 & Sharifi *et al.*, 2001, 2009, 2011), into the antimicrobial activity of whole gums and their fractions related to mastic, some high molecular weight polymers have been isolated from these naturally occurring gums. In particular, we describe their characterisation by Gel Permeation Chromatography (GPC), and by ^13^C NMR spectroscopy, and relate their high molecular weight and functional group characteristics to their antimicrobial activity. By comparison of the ‘kill’ kinetics of the natural polymers, and with those of some high molecular weight synthetic polymers based on polyvinyl alcohol, we propose and validate a mode of action for this antimicrobial behaviour.

## 2. Experimental

Mastic gum was purchased from Sigma Aldrich (Sydney Australia), composite kurdica gum was collected from Kurdistan, Iran; composite mutica from Shiraz, Iran and composite cabolica from Cabool, Afghanistan. The polymeric fractions of the gums were isolated by dissolving the gums in dichloromethane and precipitating with methanol as described elsewhere (Van den Berg, K., 1998, Ebrahimi, *et al.*, 2008, Sharifi & Hazell, 2009). The collected polymer was then analysed by ^13^C nuclear magnetic resonance (NMR) to confirm the structure. NMR spectra were obtained at UNSW using a Bruker DMX-500 operating at 500.13 and 125.75 MHz for ^1^H and ^13^C respectively, with a 5 mm triple inverse probe, from samples of the polymers dissolved in deuteriochloroform, CDCl_3_, with the exception of cabolica, for which N,N-dimethylformamide-*d*_7_, was used because of the poor solubility in CDCl_3_. ^13^C spectra were acquired with ^1^H decoupling, and referenced to the central peak in CDCl_3_, 77 ppm.

Molecular weights were determined using GPC comprised of a NSI-33R Milton Roy Minipump (Riviera Beach FL), a Rheodyne, Model 7010 injector with a 100-μl sample loop, a Gilson (Middleton, WI) 112 UV/Vis detector, and a Waters Associates–Millipore (Milton, MA) R401 Differential Refractometer that was coupled to a Kipp and Zonen recorder. A Viscotek T-Column 300mm L×7.8mm ID from Malvern Innovative Solution was connected between the injector and the detectors and the packing material was a porous styrene divinylbenzene copolymer. Flow rate was 2.0 mL/min and the chart recorder speed was 1 cm/min. Tetrahydrofuran, THF, was used as used as the eluent and all measurements were done at room temperature. UV spectra were measured at room temperature using a Varian Cary 50 and processed with the Cary WinUV software.

### 2.1 Synthesis of Polymyrcene

β-myrcene (4.74 g), cyclohexane (7.7 g), and sec-butyl lithium (400 µL) were incubated at 60 ºC for 3.5 h. Sharifi & Hazell, Newmark & Majumdar to yield *cis*-1,4-poly-β-myrcene (Figure. 1).

The poly-β-myrcene was collected for molecular weight determination by GPC, NMR analysis (Table 1) and antimicrobial activity as described below.

**Table 1:** Polymeric fractions of the *Pistacia* gums and synthetic polymyrcene analysed by GPC

Source of Gum	High Molecular Weight Fraction(Polymer) %	Whole Gum (Total weight) %	Number average molecular weight (Mn)	Weight average molecular weight (Mw)	Distribution of molecular weight
*P. a. kurdica* (kurdica gum)	13.8	100	1023	11876	250-55000
*P. a. mutica* (mutica gum)	19.4	100	906	3584	250-50000
*P. a. cabolica* (cabolica gum)	20.0	100	682	3611	250-30000
*P. lentiscus* (mastic gum)	35.2	100	20000	80000	50000-130000
*cis*-1,4-poly-β-myrcene	N/A	N/A	221823	405703	50000-500000

### 2.2 Synthesis of co-poly (vinyl-p-benzoate) (CPVPB) - A Novel Anti-microbial Polymer

**Figure 2:**
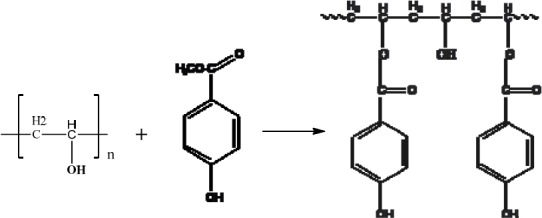
Reaction of Polyvinyl Alcohol, 2, with Methyl p-hydroxybenzoate, 3, to give Co-poly (vinyl-p-benzoate) (CPVPB), 4

Four polyvinyl alcohols, PVA’s (see 2, Figure 2) with molecular weights ranging from 13,000-146,000 were chosen to synthesise novel soluble antimicrobial polymers, in order to test the hypothesis of the relationship between molecular weight of the polymer and anti-microbial activity. Methyl p-hydroxybenzoate (see 3, Figure 2) was chosen to be incorporated into the structure of the polymer (see 4, Figure 2) on the basis of its use as a food preservative.

Two methods were used to incorporate the p-hydroxybenzoate through the ester linkage into the PVA’s: either direct esterification, or transesterification with the methyl benzoate, 3. Both methods, together with the level of incorporation, influenced the structure of the polymer product, which in turn may have influence on the antimicrobial activity against a range of Gram-positive and Gram-negative bacteria. As the incorporation of p-hydroxybenzoate using transesterification was greater than from direct esterification, the product of transesterification was chosen for detailed studies.

#### 2.2.1 Transesterification

12 g of polyvinyl alcohol was suspended in a solution of 400 mL dry dimethylformamide (DMF), 100 mL of dry benzene (used to remove small quantities of water through its azeotrope), 15 mL triethanolamine (catalyst) and 2 g of methyl p-hydroxybenzoate. The mixture was heated under reflux using a Dean and Stark apparatus, removing 125 mL of distillate, during which time the polymer was had dissolved. A further 10 g of methyl p-hydroxybenzoate was dissolved in 25 mL of dry DMF and added through the condenser and the heating at reflux continued. Approximately 150 mL of distillate was removed slowly over a period of 6 h and then the heating was continued for a further 15 h. The resulting solution was allowed to cool to room temperature, and a semi-transparent gel was produced.

The gel was broken up and suspended in 1.25 L of absolute alcohol. The mixture was stirred for 2 h until the DMF, triethanolamine and un-reacted methyl p-hydroxybenzoate had been leached from the product. The resulting polymer was filtered through Whatman grade 40/8μm (Sigma-Aldrich) and dissolved in 300 mL of milli-Q water, then was precipitated into 1.5 L of acetone and stirred to remove remaining contaminants. Any large polymer “lumps” were broken up with a glass rod, resulting in a white and stringy product. After filtration through Whatman grade 40/8 μm filter paper, the solid product was then re-suspended in 400 mL of absolute ethanol and stirred for 1h. This solid polymer was again filtered off through Whatman grade 40/8μm and then dissolved in 300 mL of distilled water.

The process of dissolution (water), precipitation (acetone), suspension (ethanol) with stirring, was repeated until no detectable Ultra Violet (UV) absorption characteristic of the p-hydroxybenzoate was detected in the supernatant ethanol.

The solid white polymer product CPVPB, 4, Figure 2, was then filtered off through Whatman grade 40/8μm and air-dried. These trans-esterification conditions were expected to result in a low level of incorporation of p-hydroxybenzoate into the polymer and thus the process returned approximately the same mass as the original polymer (12 g).

#### 2.2.2 Purification of the CPVPB polymer

Any remaining low molecular weight impurities under MW 124,000 (including those from the solvents) were removed from the polymer by dialysis against water using high molecular weight cut-off dialysis tubing consisting of a cellulose membrane (DTCM), prior to antimicrobial trials. This was to ensure that any positive result is indeed due to activity of the polymer and not the product of any low molecular weight contaminants.

Some DTCM was softened in distilled water over night. One end of the tube was well sealed, product added and the other end sealed. The tube containing the product was then placed in a large measuring cylinder filled with distilled water, which was slowly stirred by a magnetic stirrer. Every half hour, small aliquots of the water were taken from the cylinder and UV absorption was measured.

No rapid increase in the UV absorbance was observed in the water soon after placing the tube into the cylinder. This was indicative of both the integrity of the tubing and the absence of a high level of contamination from low molecular weight compounds.

The dialysis water was changed several times until no UV absorption could be detected. The solution within the dialysis tube was removed and then dried for determining the level of incorporation of p-hydroxybenzoate by UV followed by antimicrobial trials. The above procedure was repeated for all four different molecular weight compounds.

#### 2.2.3 Mole fraction of incorporated p-hydroxybenzoate

Methyl-p-hydroxybenzoate absorbs at 255 nm at neutral pH and shifts to 285 nm in basic conditions. The polymer-ester exhibited maximum absorbance at 257 nm in neutral aqueous conditions and 299.9 nm under basic conditions; slightly different from the methyl ester in neutral condition and markedly different under basic condition.

As the molar absorption coefficient of this ester was not known, the mole fraction of p-hydroxybenzoate incorporation was determined by hydrolysing the p-hydroxybenzoate from the polymer in an alkaline medium and measuring the mole fraction of p-hydroxybenzoic acid (di-salt) in the hydrolysate (maximum absorption at 249 nm in neutral conditions and 280 nm under alkaline conditions).

A solution of the polymer in water (310 mg/L) had a maximum UV absorbance at 257 nm. When diluted with 0.1 M NaOH, the maximum shifted to 299.9 nm (theoretical absorbance maximum is at 300.5 nm). To liberate p-hydroxybenzoic acid (as its di-sodium salt), the alkaline solution was warmed to 70°C for 5 min and then cooled to 5°C. The hydrolysis was quantitative and the maximum absorbance at 280 nm was measured.

The hydrolysate (alkaline solution) was acidified with sufficient acid solution (0.2 M HCl) and its UV spectrum was taken at 249 nm to measure the concentration of p-hydroxybenzoic acid present in its protonated form.

Using the calculated molar extinction coefficient, the concentration of the p-hyroxybenzoic acid incorporated into polymer was calculated (Table 2).

**Table 2:** Mole fractions of p-hydroxybenzoic acid incorporated into the polymer

Molecular weight	Mole fraction p-hydroxybenzoate (%)
13000-23000	0.15
31000-50000	0.14
50000-85000	0.13
85000-146000	0.12

### 2.3 Antimicrobial activity of the natural and synthetic polymers

#### 2.3.1 MIC and MBC

The Minimum Inhibitory Concentration (MIC) and Minimum Bacterial Concentration (MBC) values were determined against nine strains of *H. pylori*. All other Gram-positive and Gram-negative bacteria tested are listed in result section, for polymer fractions of the gums, for synthetic poly-myrcene and CPVPB, using a broth micro-dilution method Ebrahimi *et al* (2008), and Sharifi *et al* (2001, 2009, 2011).

#### 2.3.2 H. pylori, strain 26695

The strain 26695 *H. pylori* was chosen for time-kill kinetic experiments as described previously Ebrahimi *et al* (2008), and Sharifi *et al* (2001, 2009, 2011). The experiments were performed with static liquid cultures containing Isosensitest broth (Oxoid) supplemented with 5% horse serum (Oxoid). The inoculum was harvested with Isosensitest broth from 36 h cultures grown on *Campylobacter* Selective Agar (CSA).

#### 2.3.3 Gram-negative and Gram-positive bacteria

A 100 mL Iso-sensitest broth (Oxoid) culture was inoculated with a 10% inoculum from an 18 h overnight Gram-positive or Gram-negative bacterial culture. The culture was allowed to grow to stationary phase and that was determined by taking the Optical Density 600 (OD600) of the culture. The inoculums were then adjusted with the culture medium to give a starting bacteria concentration of 1.00 × 1.00 E + 08 Ebrahimi *et al* (2008), and Sharifi *et al* (2001, 2009, 2011).

Each culture was incubated for 2 h to allow recovery of the bacteria before the polymers, *cis*-1,4-β-polymyrcene and their partially oxidised forms, 4-hydroxybenzoic acid and CPVPB were added at their respective MIC and 5 × MIC concentrations. Control cultures at MIC and 5 × MIC containing appropriate solvent were also performed Ebrahimi *et al* (2008), and Sharifi *et al* (2001, 2009, 2011).

## 3. Results and Discussion

**Figure 3:**
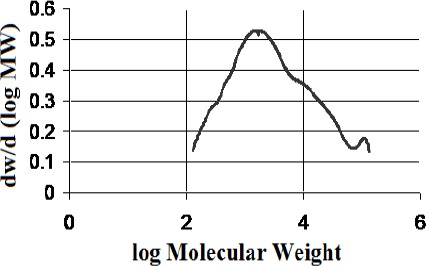
Molecular weight distribution of polymer from “kurdica gum”(Mn 1023, Mw 11876)

**Figure 4:**
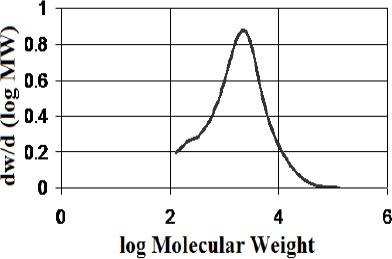
Molecular weight distribution of the polymer from “mutica gum”(Mn 906, Mw 3584)

**Figure 5:**
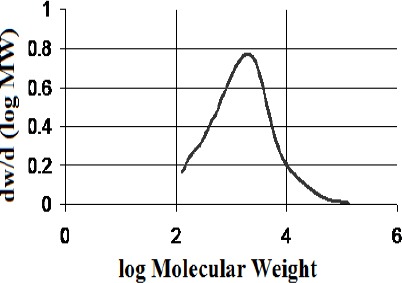
Molecular weight distribution of the POLYMER from “cabolica gum”(Mn 682, Mw 3611)

**Figure 6:**
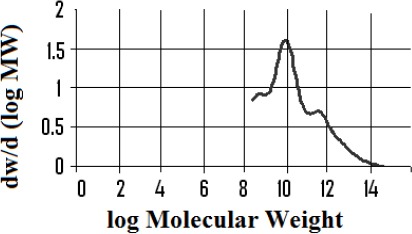
Molecular weight distribution of the polymer fraction from “mastic gum” (Mn 80,000, Mw above 120,000)

**Figure 7:**
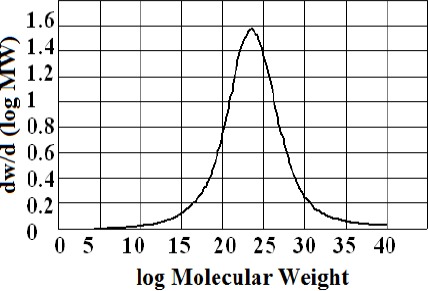
Molecular weight distribution of synthetic polymyrcene (Mn 221823, Mw 405703)

Isolation of the polymeric/high molecular weight fractions returned mass fractions of 13.8 % for the kurdica gum, 19.4% for mutica gum, 20.0% for cabolica gum and 35.2% for mastic gum. The number average molecular weight, weight average molecular weight and the distribution of molecular weight of these fractions as well as synthetic *cis*-1,4-poly-β-myrcene are shown in Table 1, which was obtained by GPC (Figures 3-7).

### 3.1 ^13^C NMR Analysis

**Figure 8:**
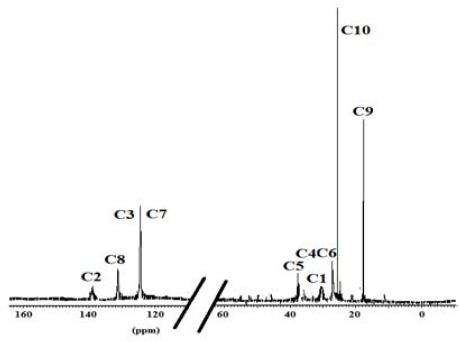
13C NMR spectrum (CDCl3 at 298 K, DMX 500) of the polymer from Mastic gum

**Figure 9:**
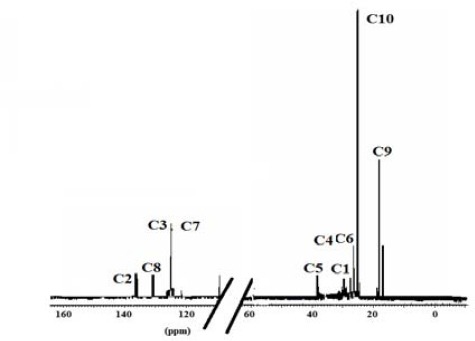
13C NMR spectrum (CDCl3 at 298 K, DMX 500) of the polymer from kurdica gum

**Figure 10:**
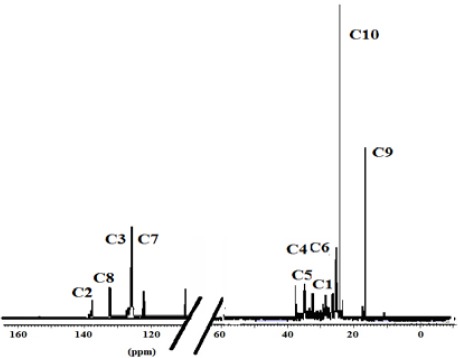
13C NMR spectrum (CDCl3 at 298 K, DMX 500) of the polymer from mutica gum

**Figure 11:**
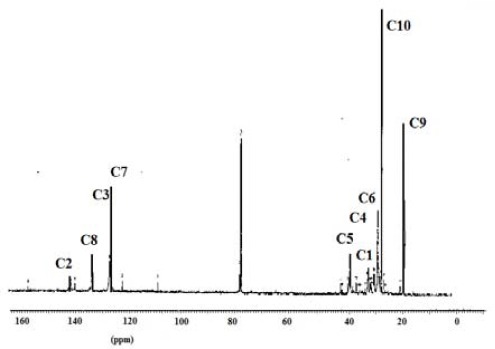
13C NMR spectrum (CDCl3 at 298 K, DMX 500) of the synthetic cis-1,4-β-polymyrcene

**Figure 12:**
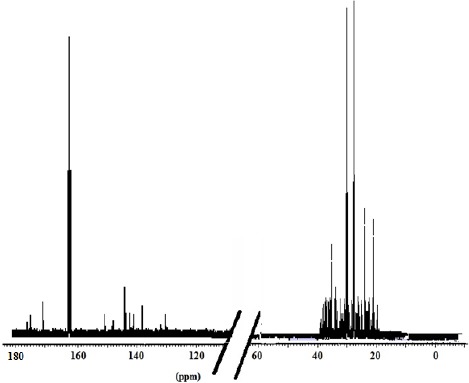
13C NMR spectrum (DMF-d7 at 298 K, DMX 500) of the cabolica gum

The ^13^C NMR spectra of polymer fractions are displayed in the Figures 8-12, and show very close similarity in constituents between *P. lentiscus* (Figure 8), *P. a. kurdica* (Figure 9), *P. a. mutica* (Figure 10) and the synthetic *cis*-1,4-poly-β-myrcene (Figure 11). However, *P. a. cabolica*’s NMR spectrum (Figure 12) is totally different, not to mention its vastly different solubility, and is clearly indicative of different structure(s). Also, it has a much lower anti-microbial activity than polymeric fractions of other gums. The NMR spectrum of mastic gum is consistent with literature Van den Berg *et al* (1998) and has been proven to be that of *cis*-1,4-poly-β-myrcene (Figure. 1). The chemical shifts of the relevant major peaks in the spectra of polymer fractions of the gums and synthetic polymyrcene listed in Table 2, and excluding cabolica gum, are assigned to *cis*-1,4-poly-β-myrcene. These chemical shifts (Table 3) are entirely consistent with the literature values Van den Berg *et al* (1998).

**Table 3:** Chemical shifts of the relevant peaks in the 13C NMR spectra of the polymer fractions of the gums and synthetic polymyrcene in CDCl3

	Synthetic Polymyrcene		Mastic gum		Kurdica gum	Mutica gum
**Assignment**	Chemical Shift		Chemical Shift		Chemical Shift	Chemical Shift
**C1**	30.73	30.56^a^	30.90	31.50^a^	30.90	30.93
**C2**	138.91	138.86^a^	38.92	139.50^a^	38.90	39.00
**C3**	124.62	124.52^a^	124.81	125.00^a^	124.78	124.70
**C4**	27.00	26.74^a^	27.96	28.10^a^	27.76	27.93
**C5**	37.73	36.85^a^	37.85	38.10^a^	37.63	37.90
**C6**	27.04	26.92^a^	27.63	27.80^a^	27.60	27.80
**C7**	124.37	124.36^a^	125.00	125.00^a^	125.05	125.10
**C8**	131.23	131.01^a^	130.93	131.80^a^	130.80	131.00
**C9**	17.57	17.64^a^	18.78	18.80^a^	18.70	19.01
**C10**	26.59	25.62^a^	26.50	26.70^a^	26.50	27.03

a Literature values from reference (6, 20).

### 3.2 Aerial Oxidation and Mastication

The susceptibility of polymyrcene to aerial oxidation was established by testing with 2,4-dinitrophenylhydrazine (2,4-DNP), to detect the presence of aldehydes and ketones, as would be expected from oxidation of the double bonds in the polymer. Thus, while no colour change was observed when the un-aerated polymer was tested with 2,4-DNP, a yellow precipitate formed with the aerated polymer sample. Likewise, a yellow precipitate was also formed when a sample of masticated gum was treated with 2,4-DNP, but no change was observed when un-masticated gums were treated with 2,4-DNP, indicating that the mastication process resulted in some level of oxidation. Lastly, the behaviour of the synthetic *cis*-1,4-poly-β-myrcene under aerobic conditions also tested positive to 2,4-DNP, forming a yellow precipitate, while the un-aerated one did not, indicating oxidation of the double bonds in the polymer.

The MIC of polymer ranged from 250 to 500 µg/mL. Upon oxidation of this fraction, its MICs shifted to a range of 125 to 250 µg/mL.

### 3.3 UV Spectroscopy

The incorporation of p-hydroxybenzoic acid into co-poly (vinyl-p-benzoate) for MW of 13000-23000, 31000-50000, 50000-85000 and 85000-146000 were measured are shown in Table 1.

### 3.4 MIC and Kill kinetics

The MIC results have been tabulated in Tables 4, 5 and 6 for polymers obtained from the gums, synthetic *cis*-1,4-poly-β-myrcene, the four co-poly(vinyl-p-benzoate) and 4-hydroxbenzoic acid against the strains of *H*. *pylori* listed in Table 4. Activities against Gram-positive and Gram-negative bacteria are listed in Table 5 and 6.

**Table 4:** MIC values of partially oxidized polymers from cis-1,4-β-polymyrcene, from Pistacia gums and polymers of CPVPB activity against strains of H. pylori (µg/mL)

Bacterial Strains	β-myrcene/Oxidised	Mastic Polymer/Oxidised Polymer	Kurdica Polymer/Oxidised Polymer	Mutica Polymer/Oxidised Polymer	Cabolica Polymer/Oxidised Polymer	Polymyrcene/Oxidised Polymyrcene	4-hydroxy-benzoic acid	CPVPB MW 13000-23000	CPVPB MW 23000-50000	CPVPB MW 50000-85000	CPVPB MW 85000-146000
26695	1000/1000	200/100	500/250	500/250	1000/1000	100/50	2000	1000	750	500	500
J99	1000/1000	250/100	250/125	250/125	1000/1000	100/50	2000	1000	750	500	500
RSB6	1000/1000	250/100	500/250	1000/500	1000/1000	100/50	2000	1000	750	500	500
P10	1000/1000	200/100	500/250	500/250	1000/1000	100/50	2000	1000	750	500	500
SS1	1000/1000	250/100	250/200	1000/500	1000/1000	100/50	2000	1000	750	500	500
SS2000	1000/1000	250/100	500/200	1000/500	1000/1000	100/50	2000	1000	750	500	500
N6	1000/1000	250/100	500/250	250/125	1000/1000	100/50	2000	1000	750	500	500
NCTC11637	1000/1000	250/100	500/250	250/125	1000/1000	100/50	2000	1000	750	500	500
RU1	1000/1000	250/100	500/250	250/125	1000/1000	100/50	2000	1000	750	500	500

**Table 5:** The MIC values of partially oxidized polymers from cis-1, 4-poly-β-myrcene, from Pistacia gums and polymers of CPVPB activity against gram negative bacteria (µg/mL)

Gram negative bacteria	β-myrcene/Oxidised	Mastic Polymer/Oxidised Polymer	Kurdica Polymer/Oxidised Polymer	Mutica Polymer/Oxidised Polymer	cabolica Polymer/Oxidised Polymer	Polymyrcene/Oxidised Polymyrcene	4-hydroxy-benzoic acid	CPVPB MW 13000-23000	CPVPB MW 23000-50000	CPVPB MW 50000-85000	CPVPB MW 85000-146000
*Escherichia coli* type 1	1000/1000	250/100	400/250	500/250	1000/1000	100/75	2000	1000	750	500	500
*Salmonella typhimurium*	1000/1000	250/100	500/200	250/125	1000/1000	100/75	2000	1000	750	500	500
*Serratia marscens*	1000/1000	250/100	500/400	1000/500	1000/1000	100/75	2000	1000	750	500	500
*Pseudomonas aeruginosa*	1000/1000	250/100	500/250	500/250	1000/1000	100/75	2000	1000	750	500	500
*Alcaligenes faecalis*	1000/1000	250/100	500/300	1000/500	1000/1000	100/75	2000	1000	750	500	500
*Enterobacter aerogenes*	1000/1000	250/100	500/200	1000/500	1000/1000	100/75	2000	1000	750	500	500
*Pseudomonas fluorescens*	1000/1000	250/100	250150	250/125	1000/1000	100/75	2000	1000	750	500	500
*Proteus vulgaris*	1000/1000	250/100	250125	250/125	1000/1000	100/75	2000	1000	750	500	500
*Porphyromonas gingivalis*	1000/1000	200/100	200/125	250/125	1000/1000	100/75	2000	1000	750	500	500

**Table 6:** The MIC values of partially oxidized polymers from cis-1,4-poly-β-myrcene, from Pistacia gums and polymers of CPVPB activity against gram positive bacteria (µg/mL)

Gram positive bacteria	β-myrcene/Oxidised	Mastic Polymer/Oxidise Polymer	Kurdica Polymer/Oxidise Polymer	Mutica Polymer/Oxidise Polymer	Cabolica Polymer/Oxidise Polymer	Polymyrcene/Oxidised Polymyrcene	4-hydroxy-benzoic acid	CPVPB MW 13000-23000	CPVPB MW 23000-50000	CPVPB MW 50000-85000	CPVPB MW 85000-146000
***Bacillus cereus***	1000/1000	1000/1000	500/250	500/250	1000/1000	1000/1000	2000	1000	750	500	500
***Staphylococcus aureus***	1000/1000	1000/1000	500/250	500/250	1000/1000	1000/1000	2000	1000	750	500	500
***Streptococcus faecalis***	1000/1000	1000/1000	500/200	500/200	1000/1000	1000/1000	2000	1000	750	500	500
***Staphylococcus epidermidis***	1000/1000	1000/1000	500/200	500/200	1000/1000	1000/1000	2000	1000	750	500	500
***Bacillus subtilis***	1000/1000	1000/1000	500/200	500/200	1000/1000	1000/1000	2000	1000	750	500	500
***Corynebacterium sp.***	1000/1000	1000/1000	500/200	500/200	1000/1000	1000/1000	2000	1000	750	500	500

### 3.5 Kill kinetics

We have reported a Generalized Multiplicative Analysis Of Variance (GEMANOVA) approach to model the complex kill kinetics data Ebrahimi *et al* (2008). The variables studied were: antibacterial agent (*β*-*myrcene*, *mastic, kurdica*, *mutica* and *cabolica*, that were labelled by Bm, S, R, T and B due to a pending patent), structure (monomer, polymer), oxidation form (oxidized, non-oxidized), concentration (1 and 5 × MIC) and bacteria (*Escherichia coli*, *Helicobacter Pylori* and *Staphylococcus aureus*). The model obtained from the aforementioned data (shown in results section) revealed the followings:

(a) The polymer fraction of *cabolica*, *kurdica* and *mutica* kill all three genera of bacteria at a concentration of 5 × MIC. Their oxidised form does not have an effect on their activity.
(b) Bacteria *E. coli* and *H. pylori* are more susceptible to the polymer form and at the higher concentration than the monomer and low concentration. *Kurdica* and *β-myrcene* are the most and least effective substances respectively for these two bacteria. Sensitivity is not affected by the oxidised form of the antibacterial substances.
(c) The monomers exhibit the same activity on the three bacteria studied. The oxidised form does not impact on the effectiveness of monomer form of antibacterial substances and greater activity is observed in higher concentrations (5xMIC).

It was demonstrated that the mass fractions of the polymers within the gums and their average molecular weight (Mn)s showed values ranging from 13.8-35.2% and 682-221823 respectively (Table 2). NMR Spectra (Figure 3-6) are indicative of very similar chemical structures with respect to the natural occurring and synthesised polymers, and, apart from cabolica gum, which requires further investigations, the polymeric fractions of the gums could be readily assigned to *cis*-1,4-poly-β-myrcene.

The MIC values and the ‘kill’ kinetics data of the polymers (naturally occurring *cis*-1,4-poly-β-myrcene, β-myrcene, and synthetic *cis*-1,4-β-poly myrcene, 4-hydrobenzoic acid and CPVPB and their antimicrobial relationship were statistically investigate (Ebrahimi, *et al.*, 2008).

The antimicrobial activities of the polymeric fractions of the gum suggest that β-myrcene, the monomer of these polymers, may also have antimicrobial activity (Ebrahimi, *et al.*, 2008). However, while knowing the structure, the question arises as to what are the active sites of these polymeric fractions and why they are more active against Gram-negative than Gram-positive bacteria, despite of the fact that the monomer has the same MIC values for Gram-positive and Gram-negative bacteria.

**Figure 13:**
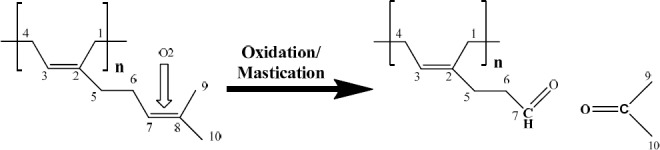
Oxidation and mastication of polymeric fraction

One of the activating factors is oxidation or mastication, as a result of which, aldehydes and ketones may be formed. Strong supporting evidence for this comes from testing the partially oxidised polymyrcene with 2,4-DNP to give a yellow precipitate i.e. a positive test. Thus oxidative activation leads to the formation of aldehydes and ketones as shown schematically in Figure 13, by oxidative cleavage of the double bond between carbons 7 and 8. The polymeric backbone will then carry the reactive aldehyde groups able to interfere with the bacterial surface, possibly augmented by the released acetone.

**Figure 14:**
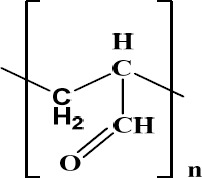
Bioactive polymer with the aldehydic functional group active against H. pylori

The antimicrobial activities of aldehydes and ketones are well known (Melrose & Keleppe, 1998). The broad-based antimicrobial properties of a polymer having the repeating polymeric unit of an aldehyde (Figure 14) forms the basis of a recent patent (Melrose & Keleppe, 1998), active against *H. pylori* (Melrose & Keleppe, 1998).

## 4. Conclusions

It has been shown that poly-myrcene as part of mastic, kurdica and mutica gum and it is masticated in use, a practice consistent with its physical form and properties which suggests a possible *in situ* oxidation of the gums, taking place in therapeutic use. The concept of polymer being “activated” through a process of oxidation generating reactive aldehyde groups, is consistent with our understanding of the mechanism of action of synthetic poly-myrcene (before and after oxidation), a model which was designed to validate the suggested mode of the action. These results are consistent with the concept that the antibacterial activity of the components of gums increase as their polarity increases, and in particular as a consequence of the presence of aldehyde, ketone and/or carboxylic acid groups within their structures, the level of these groups potentially increasing as a consequence of oxidation *in situ*. They are also consistent with higher activity being demonstrated by higher molecular weight polymer, in which CPVPB was synthesized to validate this hypothesis.

The MIC data of these polymeric compounds were not consistent with contemporary antibiotics but were rather more consistent with topical disinfectant-like compounds. This work has provided insights into natural mechanisms for activating polymeric components found in the gums of *Pistacia* sp., for increasing antimicrobial activity as well as processes for incorporating active functional groups into polymers.

## References

[ref1] Al-Habbal M. J, Al-Habbal Z, Huwez F. U (1984). A double-blind controlled clinical trial of mastic and placebo in the treatment of duodenal ulcer. J Clin Exp Pharm Physiol.

[ref2] Al-Said M. S, Ageel A. M, Parmer N. S (1986). Evaluation of Mastic, a Crude Drug obtained from Pistachio lentiscus for Gastric and Duodenal Anti-Ulcer Activity. J. Ethnopharmacol.

[ref3] Ali-Shtayeh M. S, Abu Ghdeib S. I (1999). Antifungal Activity of Plant Extracts Against Dermatophytes. Mycoses.

[ref4] Batz H. G, Ringsdorf H, Ritter H (1974). Pharmacologically active polymer. Die Makromolekulare Chemie.

[ref5] Cloete T. E (2003). Resistance mechanism of bacteria to antimicrobial compounds. International Biodeterioration and Biodegradation.

[ref6] Ebrahimi D, Sharifi M. S, Hazell S. L (2008). Generalized multiplicative analysis of variance of kill kinetics data of antibacterial agents. Chemometrics and Intelligent Laboratory Systems.

[ref7] Huwez F. U, Al-Habbal M. J (1986). Mastic in treatment of benign gastric ulcers. Gastroenterol Japan.

[ref8] Kalyon B. D, Olgun U (2001). Antibacterial efficacy of triclosan-incorporated polymers. American Journal of Infection Control.

[ref9] Kerr A, Smith M. J, Cowling M. J (2001). The biofouling resistant properties of six transparent polymers with and without pre-treatment by two antimicrobial solutions. Materials and Design.

[ref10] Maron P, Bono L, Leone E (2001). Bactericidal Activity of *Pictacia lentiscus* Mastic Gum against Helicobacter pylori. J. Chemother.

[ref11] Melrose G. J. H, Keleppe C. M, Langley W. J (1998). Biostatic and biocidal compositions.

[ref12] Newmark R. A, Majumdar R. N (1998). ^13^C-NMR Spectra of *cis*-Polymyrcene and *cis*-Polyfarnesene. Journal of Polymer Science.

[ref13] Paraschos S, Magiatis P, Mitakou S (2007). *In Vitro* and *In Vivo* Activities of Chios Mastic Gum Extracts and Constituents against *Helicobacter pylori*. Antimicrob. Agents Chemother.

[ref14] Sakagami H, Kishino K, Kobayashi M (2009). Selective Antibacterial and Apoptosis-modulating Activities of Mastic. In Vivo.

[ref15] Sharifi M. S, Hazell S. L (2009). Fractionation of Mastic Gum in Relation to Antimicrobial Activity. Pharmaceuticals.

[ref16] Sharifi M. S, Hazell S. L (2011). GC-MS Analysis and Antimicrobial activity of the essential oil of the trunk exudates from Pistacia atlantica kurdica. Journal of Pharmaceutical Sciences and Research.

[ref17] Sharifi M. S, Vagg W, Hazell S. L (2001). Characterization of mastic gum in relation to anti-Helicobacter pylori activity. Journal of World Chemistry.

[ref18] Smith A. W (2005). Biofilms and antibiotic therapy: Is there a role for combating bacterial resistance by the use of novel drug delivery systems?. Advanced Drug Delivery Reviews.

[ref19] Van den Berg K, Van der Horst J, Boon J (1998). Cis-1,4-poly-ß-myrcene;the structure of the polymeric fraction of mastic resin (*Pistachia lentiscus L*.) elucidated. Tetrahedron Letters.

[ref20] Zhou L, Satoh K, Takahashi K (2009). Re-evaluation of Anti-inflammatory Activity of Mastic Using Activated Macrophages. In Vivo.

